# Single-nucleus transcriptomics of epicardial adipose tissue from female pigs reveals effects of exercise training on resident innate and adaptive immune cells

**DOI:** 10.1186/s12964-024-01587-w

**Published:** 2024-04-26

**Authors:** Irshad Ahmad, Shreyan Gupta, Patricia Faulkner, Destiny Mullens, Micah Thomas, Sharanee P. Sytha, Ivan Ivanov, James J. Cai, Cristine L. Heaps, Annie E. Newell-Fugate

**Affiliations:** 1 Department of Veterinary Physiology and Pharmacology, School of Veterinary Medicine and Biomedical Sciences, Texas A&M University, College Station, TX USA; 2 Department of Veterinary Integrative Biosciences, School of Veterinary Medicine and Biomedical Sciences, Texas A&M University, College Station, TX USA

**Keywords:** Epicardial adipose tissue, Aerobic exercise, Swine, Immune cells, Coronary artery disease

## Abstract

**Background:**

Coronary artery disease (CAD) is a leading cause of death in women. Epicardial adipose tissue (EAT) secretes cytokines to modulate coronary artery function, and the release of fatty acids from EAT serves as a readily available energy source for cardiomyocytes. However, despite having beneficial functions, excessive amounts of EAT can cause the secretion of proinflammatory molecules that increase the instability of atherosclerotic plaques and contribute to CAD progression. Although exercise mitigates CAD, the mechanisms by which exercise impacts EAT are unknown. The Yucatan pig is an excellent translational model for the effects of exercise on cardiac function. Therefore, we sought to determine if chronic aerobic exercise promotes an anti-inflammatory microenvironment in EAT from female Yucatan pigs.

**Methods:**

Sexually mature, female Yucatan pigs (*n* = 7 total) were assigned to sedentary (Sed, *n* = 3) or exercise (Ex, *n* = 4) treatments, and coronary arteries were occluded (O) with an ameroid to mimic CAD or remained non-occluded (N). EAT was collected for bulk (*n* = 7 total) and single nucleus transcriptomic sequencing (*n* = 2 total, 1 per exercise treatment).

**Results:**

Based on the bulk transcriptomic analysis, exercise upregulated S100 family, G-protein coupled receptor, and CREB signaling in neurons canonical pathways in EAT. The top networks in EAT affected by exercise as measured by bulk RNA sequencing were SRC kinase family, fibroblast growth factor receptor, Jak-Stat, and vascular endothelial growth factor. Single nucleus transcriptomic analysis revealed that exercise increased the interaction between immune, endothelial, and mesenchymal cells in the insulin-like growth factor pathway and between endothelial and other cell types in the platelet endothelial cell adhesion molecule 1 pathway. Sub-clustering revealed nine cell types in EAT, with fibroblast and macrophage populations predominant in O-Ex EAT and T cell populations predominant in N-Ex EAT. Unlike the findings for exercise alone as a treatment, there were not increased interactions between endothelial and mesenchymal cells in O-Ex EAT. Coronary artery occlusion impacted the most genes in T cells and endothelial cells. Genes related to fatty acid metabolism were the most highly upregulated in non-immune cells from O-Ex EAT. Sub-clustering of endothelial cells revealed that N-Ex EAT separated from other treatments.

**Conclusions:**

According to bulk transcriptomics, exercise upregulated pathways and networks related to growth factors and immune cell communication. Based on single nucleus transcriptomics, aerobic exercise increased cell-to-cell interaction amongst immune, mesenchymal, and endothelial cells in female EAT. Yet, exercise was minimally effective at reversing alterations in gene expression in endothelial and mesenchymal cells in EAT surrounding occluded arteries. These findings lay the foundation for future work focused on the impact of exercise on cell types in EAT.

**Supplementary Information:**

The online version contains supplementary material available at 10.1186/s12964-024-01587-w.

## Background

Coronary artery disease (CAD) is the leading cause of death for women in the United States (U.S.) [1]. Despite the large morbidity and mortality of CAD in American women, there is a general lack of awareness of both its impact on women’s health and the distinct sex-related disparities associated with this disease [2]. Thus, there is a critical need to advance knowledge focused on the pathogenesis and management of CAD in women. In humans, 80% of the heart surface area is covered by epicardial adipose tissue (EAT), which secretes cytokines to modulate physiological and pathophysiological processes in the coronary arteries and myocardium [3, 4]. Moreover, EAT is involved in the breakdown of fatty acids, serving as a local energy source for cardiomyocytes during periods of increased energy demand [5]. Despite EAT’s beneficial functions, increased EAT is detrimental to both the structure and function of the heart [6]. The altered secretion of adipokines and proinflammatory molecules from EAT causes increased instability of atherosclerotic plaques [7–10]. Thickened EAT also is associated with atrial enlargement, impaired diastolic filling, elevated myocardial lipid content and lipotoxic injury to cardiomyocytes, and cardiac remodeling [6]. Furthermore, myocardial infiltration with epicardial adipocytes and the release of cytokines into the myocardial layer results in tissue inflammation and cardiac muscle dysfunction [11]. Thus, the imbalance between the protective and harmful effects of EAT contributes to CAD progression.

**Fig. 1:**
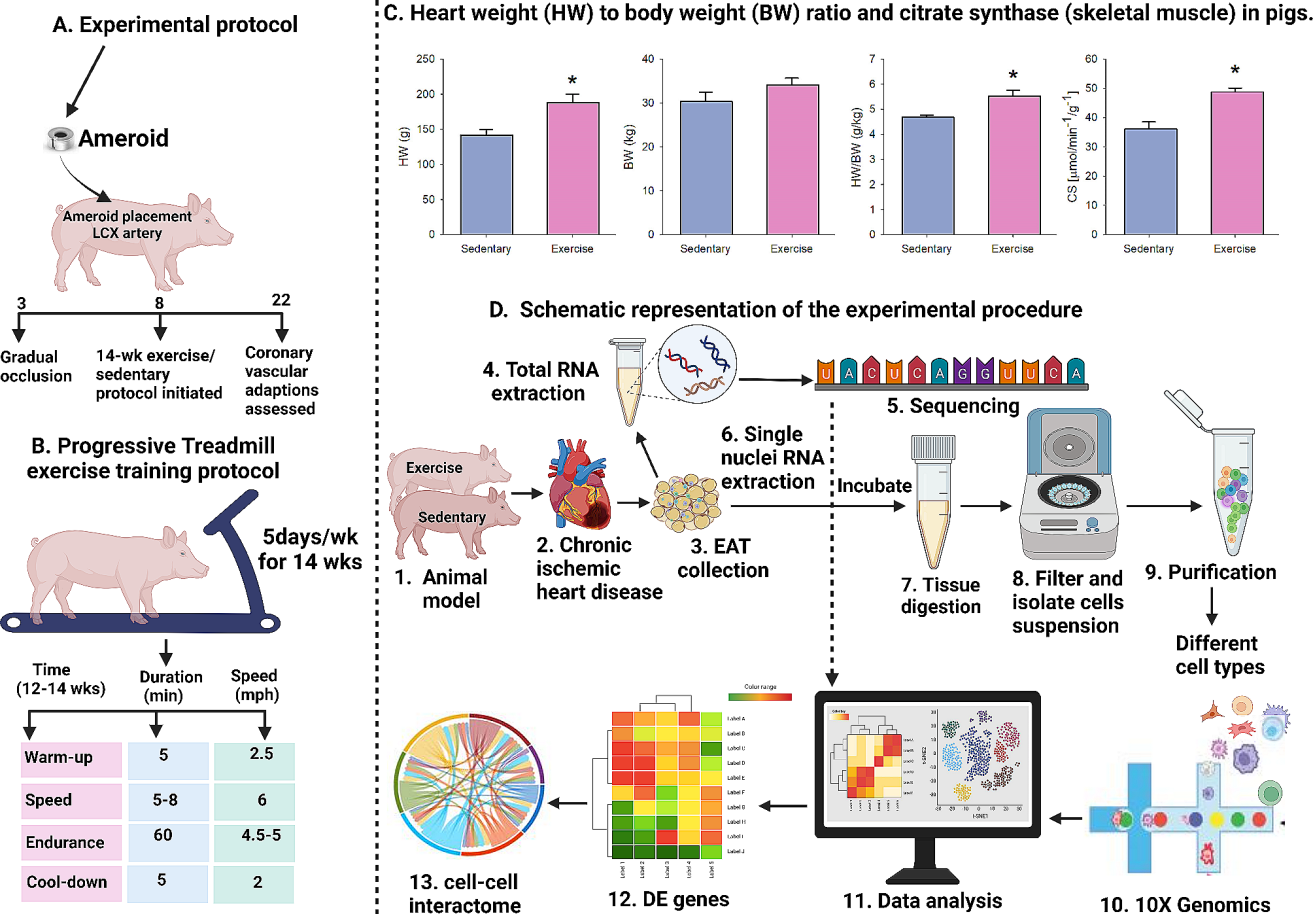
Schematic representation of the experimental protocol followed during the experiment. (**A**) Overall animal study design (**B**) exercise training regimen (**C**) Heart to body weight ratio and citrate synthase in female pigs at the end of the experiment (**D**) Bulk and single nuclei RNA extraction and computational analysis. This image was created using BioRender. CS: citrate synthase; HW: heart weight; BW: body weight; LCX: left circumflex coronary artery; EAT: epicardial adipose tissue; DE: differentially expressed. *n* = 3 per treatment group; * *p* < 0.05

Increased physical activity is recommended for the prevention and secondary treatment of CAD. Aerobic exercise mitigates CAD via enhanced cardiac function and vascular adaptations, increasing tissue blood flow to meet metabolic demands [12]. Furthermore, aerobic exercise reduces EAT volume in women [13–15]. Yet, how aerobic exercise modulates molecular mechanisms in EAT is unknown. In this study, we dissected the opposing effects of exercise and sedentary lifestyle on EAT at both the tissue and single-cell level in female Yucatan miniature swine with chronic ischemic heart disease (Fig. 1A, B, D). For this research, we utilized cardiac tissue from a translational porcine model of chronic coronary artery occlusion (Fig. 1) and exercise training. This porcine model is highly clinically relevant because it replicates many human adaptations to ischemic heart disease and exercise training [16, 17]. Furthermore, the pig is an excellent model of progressive coronary artery occlusion because both porcine and human hearts demonstrate few innate collateral vessels and the growth of new vessels typically occurs as an extensive network of functionally significant collaterals in the endocardial and mid myocardial layers [16, 17]. The impact of aerobic exercise on gene expression in EAT was investigated with bulk RNA-sequencing (RNA-seq), revealing significant alterations in growth factor and immune cell signaling pathways as well as estrogen signaling. Single-nuclei transcriptome (snRNA-seq) analysis of EAT further unveiled diverse molecular signatures for both non-immune and immune cells, along with tissue-level gene and pathway modifications due to exercise. This study highlighted the influence of aerobic exercise on intercellular communication pathways, such as insulin-like growth factor (IGF) and platelet endothelial cell adhesion molecule 1 (PECAM1). Furthermore, differential gene expression (DGE) analysis for single nuclei transcriptomics revealed that genes related to cell adhesion, migration, fatty acid metabolism, and angiogenesis were modulated in non-immune and immune cells, depending on exercise status. This study provides valuable insights into the molecular and cellular changes induced in EAT by aerobic exercise in the context of ischemic heart disease in female pigs.

## Methods

### Experimental animals and surgical instrumentation

Animal protocols were approved by the Texas A&M University Institutional Animal Care and Use Committee and conformed to the National Institutes of Health (NIH) “Guide for Care and Use of Laboratory Animals, 8th edition,” revised 2011). Adult Yucatan miniature swine (6–7 months of age) were surgically instrumented with an ameroid constrictor around the proximal left circumflex coronary artery (see Supplemental Methods).

### Sedentary and exercise protocols

Exercise-trained pigs underwent a progressive treadmill exercise training program, 5 days/week for 14 weeks, as described previously [18]. Speed and duration of the exercise training sessions were progressively increased so that during the last three weeks of training, animals ran at 4-5.5 mph for 60 min and at 6 mph for 5–15 min. The grade of the treadmill was maintained at 0% throughout the exercise bouts. The endurance portion of the treadmill run was performed at ∼70% maximal O2 consumption, based on O2 consumption data obtained from Yucatan miniature swine by McAllister and colleagues [19]. The progressive nature of the exercise regimen was dependent upon the tolerance of each pig, and therefore, the ranges of speed and duration presented represent differing abilities of the animals. Exercise-trained and sedentary swine were fed once daily immediately after the exercise training session to serve as positive reinforcement for the exercise bout, with water provided *ad libitum*. Sedentary pigs maintained normal activity in their pens throughout the experimental protocol. The effectiveness of the exercise training regimen was determined by comparing heart-to-body weight ratio and skeletal muscle citrate synthase activity. Animal heart weight, body weight, heart-to-body weight ratio, and skeletal muscle citrate synthase values were compared using a Student’s t-test. Data are presented as mean ± SEM, and *n* values in parentheses reflect the number of animals studied.

### Isolation of coronary epicardial adipose tissue

Following completion of the 14-week exercise training or sedentary protocol, pigs were humanely euthanized, and epicardial perivascular fat was sectioned from both the non-occluded left anterior descending coronary artery and the collateral-dependent left circumflex coronary artery. Perivascular fat was snap-frozen in liquid nitrogen and subsequently stored at -80 °C for later analysis. Subsequent visual inspection of the ameroid occluder during dissection of the left circumflex artery under a dissection microscope during dissection of the left circumflex artery indicated 100% occlusion in all pigs used in this study.

### Bulk transcriptomics

EAT (occluded and non-occluded tissue pooled) from exercise-trained (*n* = 4) and sedentary (*n* = 3) female pigs had total RNA extracted with TRIzol® (Thermofisher Scientific, Waltham, MA). Total RNA was quantified (Nanodrop 3300, Thermofisher, Wilmington, DE) followed by bioanalysis (Agilent 2100 Bioanalyzer, Agilent Technologies, Inc., Santa Clara, CA) for RNA quantity and quality. RNA integrity scores fell within the following range: 6.2–9.1. RNA sequencing libraries were prepared using the Illumina TruSeq Stranded Total RNA preparation kit (Illumina Inc., San Diego, CA) at the Molecular Genomics core at Texas A&M. Sequencing was performed on the Illumina NovaSeq 6000 on an S4 2 × 150 flow cell at the North Texas Genome Center (University of Texas – Arlington, Arlington, TX).

### Single-nucleus RNA sequencing

Isolation of EAT nuclei was performed following the “Daughter of Frankenstein protocol for nuclei isolation from fresh and frozen tissues using OptiPrep continuous gradient V.2” [20]. Nuclei were resuspended in 0.1% BSA in PBS and immediately processed for the generation of single-nuclei RNA libraries using the microdroplet-based RNA. Nuclei samples were diluted, if necessary, to a target concentration of between 500 and 1,500 nuclei/µL and used for single nuclei RNA sequencing library preparation (see Supplemental Methods).

### Bioinformatic analysis – bulk sequencing

Raw bulk RNA sequencing data was quality control checked with FastQC v0.11.9. Mapping was performed using Bowtie2 version 2.3.5.1 (64-bit) with default settings [21]. Counting was performed using htseq-count version 2.0.2 [22]. Data was normalized using the upper quartile method. Differential gene expression (DGE) was performed in R using edgeR version 3.34.1 [23–26] and limma 3.48.3 [27]. Differentially expressed genes with raw *p* values < 0.05 (Log Fold Change > 1) were imported into Qiagen Ingenuity Pathway Analysis software version 107,193,442 build ing_neptunite. There were 533 genes (247 downregulated and 286 upregulated) for the analysis. Expression analysis was conducted on the differentially expressed genes, and the following were generated: canonical pathway analysis, casual networks, and upstream regulators.

### Bioinformatic analysis – single nuclei sequencing

UMI count matrices for each single-cell sample were generated by aligning reads to the genome (Landrace_pig_v1 (GCA_001700215.1) using 10X Genomics Cell Ranger software. The DGE analysis was performed using edgeR package in R [23–26]. Low-quality cells and under-expressed genes were excluded. The *p* values were False Discovery Rate (FDR) corrected for multiple testing using Benjamini–Hochberg procedure. The top 10 significant differentially expressed genes (*p* < 0.05 and Average Log Fold Change > 1) for each treatment group were plotted in dot plots for each cell type and treatment group combination. The total number of upregulated and downregulated differentially expressed genes for each combination of treatment group and cell type was plotted as a heatmap. The functional enrichment of the top 100 DEGs was carried out using Enrichr (https://maayanlab.cloud/Enrichr/) to identify key enriched pathways (*p* < 0.01). The scType database was used to further cluster and annotate the mesenchymal cells [28]. An unknown cluster of cells was removed from the study as no significant markers corresponding to the atlas were found, resulting in 20,655 genes across 24,382 cells for further analysis.

For within-and cross-tissue communication prediction, UMI count matrices and cell type/state assignment were exported for each cell as two input files for the CellChat R package. CellChat is a publicly available repository of curated receptors, ligands, and their interactions. CellChat R Package was used to infer cell-cell communication within and across cell types using the CellChatDB database. For each cell type, significantly differentially overexpressed ligand-receptor pairs were identified. Next, the communication likelihood was computed using the average expression values of a ligand in one cell type and that of a receptor/cofactor in another cell type. Significant interactions were pruned by permutation tests (*p* < 0.05). An intercellular communication network was generated along with communication probabilities that measure the strength of relationships between each ligand-receptor pair. The communication network for each signaling pathway was generated by summing up all corresponding ligand-receptor interactions for that pathway. The CellChatDB was subset to specifically identify paracrine, endocrine and juxtacrine signaling pathways.

## Results

### Fourteen-week aerobic exercise training regimen increases the heart-to-body weight ratio and skeletal muscle citrate synthase activity in female pigs

Despite no difference in body weight between exercise-trained and sedentary pigs, the heart-to-body weight ratio was larger in exercise-trained pigs compared with sedentary pigs, which indicates exercise training-induced cardiac hypertrophy (Fig. 1C). Evaluation of skeletal muscle citrate synthase activity after completing the sedentary and exercise-training regimens revealed increased oxidative enzyme activity of the medial head of the triceps brachii muscle of exercise-trained swine (Fig. 1C). Taken together, these data demonstrate the effectiveness of the 14-week aerobic exercise-training program in producing adaptations indicative of the exercise-trained state.

### Aerobic exercise upregulates pathways and processes related to cellular migration, proliferation, and differentiation in EAT

**Fig. 2:**
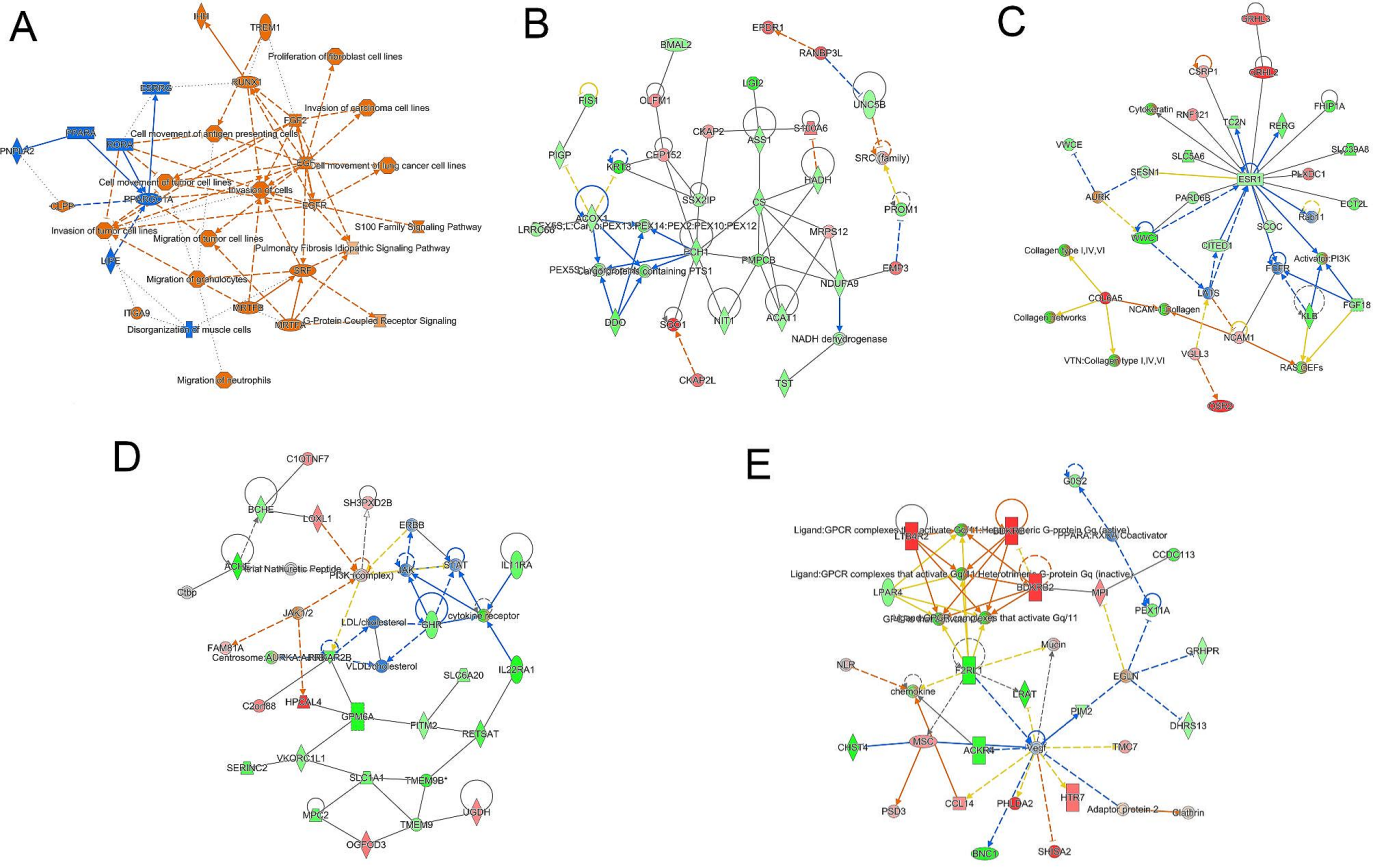
Pathways and diseases in whole epicardial adipose tissue of female pigs affected by exercise. (**A**) Graphical summary of genes and processes impacted by exercise. Orange: predicted upregulation; Blue: predicted downregulation; White: no effect. (**B**) SRC family network in whole EAT impacted by exercise (**C**) FGFR-ESR1 network in whole EAT impacted by exercise (**D**) JAK-STAT network in whole EAT impacted by exercise. (**E**) VEGF-F2RL1 network in whole EAT affected by exercise. Green: decreased gene expression; Red: increased gene expression; Blue: predicted gene inhibition; Orange: predicted gene activation

We performed RNA-seq on EAT from exercise-trained and sedentary female pigs. Canonical pathways upregulated in order of most to least significance were (overlap percentage): S100 family (5.2%), G-protein coupled receptor (GPCR) signaling (5.1%), pulmonary fibrosis idiopathic signaling pathway (6.4%), molecular mechanisms of cancer (4.6%), cAMP response element binding protein (CREB) signaling in neurons (4.9%). Pathway analysis based on z score and *p* value predicted that exercise would inhibit (negative z score) the activity of estrogen-related receptor gamma (*ESRRG*), peroxisome proliferator-activated receptor alpha (*PPARA*), patatin-like phospholipase domain containing 2 (*PNPLA2*), RAR related orphan receptor A (*RORA*), PPARG coactivator 1 alpha (*PPARCG1A*), lipase E hormone-sensitive type (*LIPE*), and inhibit the disorganization of muscle cells (Fig. 2A). Processes predicted to be activated in response to exercise were cell movement of antigen-presenting cells, proliferation of fibroblast cell lines, invasion of carcinoma cell lines, cell movement, invasion and migration of tumor cell lines, cell movement of lung cancer cell lines, and migration of neutrophils and granulocytes (Fig. 2A). Activation (positive z score) was predicted for the following genes: fibroblast growth factor 2 (*FGF2*), epithelial growth factor (*EGF*), epithelial growth factor receptor (*EGFR*), serum response factor (*SRF*), RUNX family transcription factor 1 (*RUNX1*), Indian hedgehog signaling molecule (*IHH*), triggering receptor expressed on myeloid cells 1 (*TREM1*), caseinolytic mitochondrial matrix peptidase proteolytic subunit (*CLPP*), integrin subunit alpha 9 (*ITGA9*), myocardin related transcription factor B (*MRTFB*), myocardin related transcription factor A (*MRTFA*) (Fig. 2A).

The top five networks differentially affected by exercise based on *p* value and number of focus molecules in order of most significant to least were: SRC family (Fig. 2B), FGFR-estrogen receptor alpha (*ESR1*) network (Fig. 2C), JAK-STAT-cholesterol network (Fig. 2D), vascular endothelial growth factor (*VEGF*)-F2R like trypsin receptor 1 (*F2RL1*) network (Fig. 2E), and Actin-Wnt network (Supplemental Table 1). The top 10 upstream regulators impacted by exercise based on *p* value (z score greater than 2 predicts activation, z score less than − 2 predicts inhibition) were: estradiol (z score − 1.028), tumor necrosis factor (TNF) (z score 2.571;), transforming growth factor beta 1 (TGFB1) ( z score 1.446), KRAS proto-oncogene GTPase (KRAS) (z score 0.396), dexamethasone (z score − 1.723), lipopolysaccharide (z score 3.192), tumor protein 53 (TP53) (z score 0.18), AT-rich interaction domain 1 A (ARID1A) (z score − 0.428), angiotensin (z score 2.742), interleukin 1 beta (IL1B) (z score 1.177) (Supplemental Table 2). The top 10 causal master regulators of networks upstream of molecules differentially impacted by exercise based on *p* value (z score greater than 2 predicts activation, z score less than − 2 predicts inhibition) were: zinc finger protein 746 (ZNF746) (z score 3.482), cell division cycle 25 C phosphatase (z score − 0.387), SET and MYND domain containing 3 (SMYD3) (z score − 0.145), micro RNA 873 (mir-873) (z score 0.711), angiotensin (z score 0.159), ulinastatin (z score − 2.534), interleukin 13 (IL13) (z score − 1.534), decitabine (z score 0.645), Ac-YVAD-CMK (z score − 1.703), and lamivudine (z score 1.873) (Supplemental Table 3).

### Aerobic exercise increases interaction between immune and mesenchymal cells in the IGF pathway and between endothelial cells, fibroblasts, and other cell types in adhesion molecule pathways

**Fig. 3:**
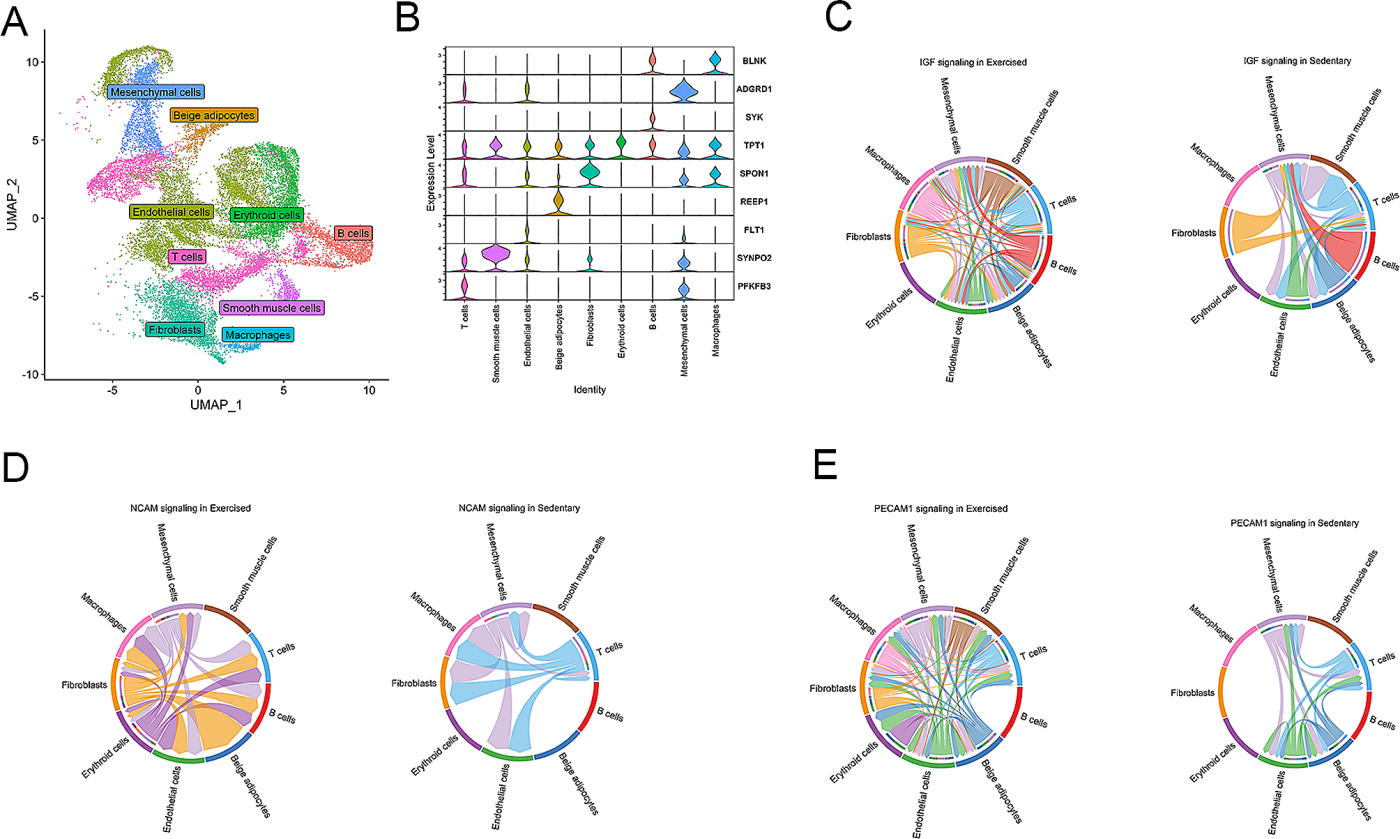
Single nuclei gene expression from epicardial adipose tissue of sedentary and exercised female pigs. (**A**) Single nuclei clustering in epicardial adipose tissue from exercise-trained and sedentary female pigs. (**B**) Expression of established marker genes for each cell type in each cluster. Circle plots representing most prominent cell-cell communications between exercise and sedentary groups in the insulin-like growth factor (IGF) (**C**), neural cell adhesion molecule (NCAM) (**D**), and (**E**) platelet endothelial cell adhesion molecule 1 (PECAM1) signaling pathways

We isolated single-nuclei suspensions from non-occluded (N) and occluded (O) regions of EAT from exercise-trained (Ex) and sedentary (Sed) swine, resulting in four experimental groups: N-Ex, O-Ex, N-Sed, and O-Sed. Single-nuclei suspensions were profiled using 10x Genomics Chromium droplet snRNA-seq. The resulting EAT single nuclei atlas had 24,382 individual nuclei that were visualized with uniform manifold approximation and projection (UMAP) and grouped into 17 clusters. We performed unsupervised clustering on the cells using the Louvain algorithm in the Seurat R package [29]. The clustering analysis revealed nine distinct clusters (Fig. 3A). These clusters were annotated using markers from the pig atlas (https://dreamapp.biomed.au.dk/pigatlas/) with specific markers linked to each cellular phenotype to identify cell types. Finally, nine unique cell types were identified based on differential expression of marker genes in the nuclei clusters (Fig. 3A, Dataset S1). Cells identified included: (1) beige adipocytes (receptor accessory protein 1 *(REEP1)*, prospero homeobox 1 *(PROX1)*, multimerin 1 (*MMRN1*), semaphorin 6 A (*SEMA6A*), par-6 family cell polarity regulator gamma (*PARD6G*); (2) endothelial cells (*PECAM1)*, fms related receptor tyrosine kinase 1 *(FLT1)*, adhesion G protein-coupled receptor *(ADGRL4)*, cysteine and tyrosine rich 1 *(CYYR1)*, dedicator of cytokinesis 9 *(DOCK9)*, scavenger receptor class A member 5 *(SCARA5)*, zinc finger and BTB domain containing 7 C *(ZBTB7C)*, phospholipase D1 *(PLD1)*, protein tyrosine phosphatase receptor type B *(PTPRB)*, CD93 molecule *(CD93)*, apolipoprotein A1 *(APOA1)*, regulator of G protein signaling 5 *(RGS5)*, receptor activity modifying protein 2 *(RAMP2)*, glycosylphosphatidylinositol anchored high density lipoprotein binding protein 1 *(GPIHBP1)*, LIM domain binding 2 *(LDB2*); (3) fibroblasts (spondin 1 (*SPON1)*, gelsolin *(GSN*); (4) mesenchymal cells (adhesion G protein-coupled receptor D1 (*ADGRD1*); (5) smooth muscle cells (synaptopodin 2 (*SYNPO2*); (6) erythroid cells (tumor protein, translationally-controlled 1 (*TPT1*); (7) macrophages (colony stimulating factor 1 receptor (*CSF1R)*, B cell linke*r (BLNK)*, amyloid beta precursor protein binding family B member 1 interacting protein *(APBB1IP)*, CD163 molecule *(CD163)*, B cell scaffold protein with ankyrin repeats 1 *(BANK1)*, stabilin 1 *(STAB1*); (8) B cells (spleen associated tyrosine kinase *(SYK)*, vav guanine nucleotide exchange factor 3 *(VAV3*), and (9) T cells (6-phosphofructo-2-kinase/fructose-2,6-biphosphatase 3 *(PFKFB3)*, IKAROS family zinc finger 3 *(IKZF3)*, protein tyrosine phosphatase receptor type c *(PTPRC)*, Src kinase associated phosphoprotein 1 *(SKAP1)*, talin 2 *(TLN2*) (Fig. 3B, Supplemental Table 4).

Intercellular communication between non-immune cells (endothelial cells, beige adipocytes, fibroblast, mesenchymal cells, smooth muscles cells and erythroid) and immune cells (macrophages, B cells, T cells) was analyzed via CellChat algorithm (Dataset S2) [30]. Aerobic exercise significantly impacted the following cell-to-cell communication signaling pathways: IGF, neural cell adhesion molecule (NCAM), and PECAM1. For IGF signaling, all immune cell classes had the largest number of interactions with endothelial and mesenchymal cells in Ex-EAT. In contrast, only B and T cells heavily communicated with mesenchymal cells in Sed-EAT (Fig. 3C). For NCAM, a glycoprotein in the immunoglobulin superfamily, the strongest communication strength was observed between fibroblast and erythroid cells with all other cell types in Ex-EAT from exercise-trained pigs. T cells and mesenchymal cells had the greatest number of communications with other cell types in Sed-EAT for NCAM signaling (Fig. 3D). PECAM1 is expressed at endothelial cell intercellular junctions and regulates leukocyte trafficking. In Ex-EAT, endothelial cells had increased interactions between all cell types, except B cells, for the PECAM1 signaling. In Sed-EAT, only T cells, endothelial cells, mesenchymal cells, and beige adipocytes interacted for PECAM1 signaling (Fig. 3E). Gene expression of factors in the IGF signaling pathway, such as *IGF1*, exhibited elevated levels in beige adipocytes, fibroblasts, and T cells. Conversely, the expression of insulin-like growth factor 1 receptor (*IGF1R*) was higher in beige adipocytes, endothelial cells, fibroblasts, and T cells of the sedentary group (Supplemental Fig. 1). In the case of the *NCAM1* gene, its expression was upregulated in fibroblasts, while significantly lower expression was observed in various other cell types. Endothelial cells and T cells displayed increased expression of fibroblast growth factor receptor 1 (*FGFR1*) (Supplemental Fig. 1). Additionally, *PECAM1* demonstrated increased expression in endothelial cells (Supplemental Fig. 1).

### Exercise alters differential gene expression in adaptive immune cells in EAT

The total number of significantly upregulated and downregulated genes in response to exercise was 183 upregulated genes in endothelial cells and 166 downregulated genes in T cells. While as during sedentary conditions, 55 genes were upregulated in mesenchymal cells and 22 genes in T cells (Supplemental Fig. 2A). In T cells, 1307 genes in non-occluded EAT and 1159 genes in occluded EAT were upregulated. In endothelial cells, 73 genes in non-occluded EAT and 103 genes in occluded EAT were upregulated (Supplemental Fig. 2B).

### Aerobic exercise upregulates cellular adhesion and metabolism genes in endothelial cells, adipocytes, smooth muscle cells, fibroblasts, and B cells

DGE analysis was performed using EdgeR between Ex-EAT and Sed-EAT. The top 10 differentially expressed genes for each cell type were sorted based on average log fold change. DGE for each cell type is in Dataset S3. For non-immune cells, DEGs included ELOVL fatty acid elongase 6 (*ELOVL6)*, acetyl-CoA carboxylase alpha *(ACACA)*, malic enzyme 1 *(ME1)*, acyl-CoA *(ACYL)*, stearoyl-CoA desaturase *(SCD)*(Supplemental Fig. 3). Enrichment analysis performed using the top 100 differentially expressed genes (*p* < 0.05) showed biosynthesis of unsaturated fatty acids, the AMPK signaling pathway, and the peroxisome proliferator-activated receptor (PPAR) signaling pathway were influenced by exercise in non-immune cells (Dataset S4; Supplemental Fig. 4). Immune cells in Sed-EAT had differentially expressed genes related to immunoregulation and basic cellular processes (i.e. FKBP prolyl isomerase 5 (*FKBP5)*, glutamate-ammonia ligase *(GLUL))* (Supplemental Fig. 5). All immune cell categories in Sed-EAT had increased gene expression of *FKBP5*, which is involved in protein folding and trafficking. *ELOVL6* was upregulated across all non-immune cell types except for mesenchymal cells in Ex-EAT (Supplemental Fig. 6). Pathway analysis showed that the JAK-STAT signaling pathway, fatty acid biosynthesis, and longevity-regulating pathway were affected in immune cells (Dataset S4).

### Comprehensive profiling of cell type-specific gene expression reveals distinct molecular signatures in EAT in response to exercise and coronary artery occlusion status

**Fig. 4:**
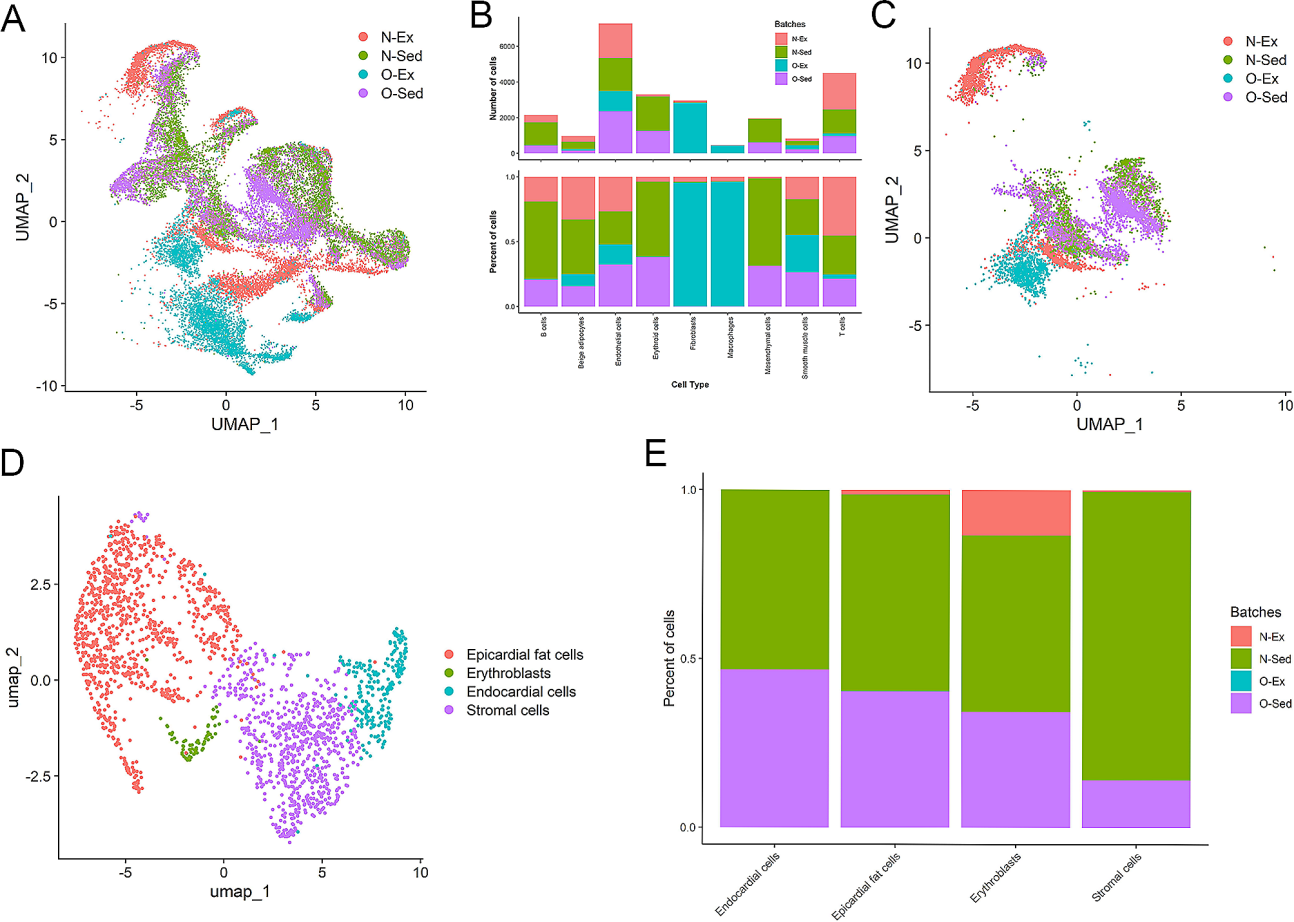
Single nuclei gene expression from epicardial adipose tissue surrounding occluded and non-occluded coronary arteries from female pigs. (**A**) Occluded and non-occluded unbiased clustering of 24,382 cells revealed nine different cell clusters. (**B**) Number and percentage of cell types in each treatment (exercise trained and sedentary) and batch-wise distribution in each occlusion*exercise treatment (occluded and non-occluded) expressed genes. (**C**) Sub-clustering of endothelial cells showed separation of N-Ex group from all other treatments. (**D**) Sub-clustering of mesenchymal cells showed four different cell types. (**E**) Percentage of each cell type in the mesenchymal subcluster by occlusion*exercise treatment. N: non-occluded; O: occluded; Ex: exercise trained; Sed: sedentary

When experimental groups were divided into N-Sed, O-Sed, N-Ex, and O-Ex, all nine cell-type clusters were found **(**Fig. 4A**).** O-Ex tissue had abundant fibroblasts, while N-Ex tissue had a large amount of T cells, beige adipocytes, and endothelial cells **(**Fig. 4B**).** O-Sed tissue had abundant endothelial cells, while N-Sed tissue had many endothelial and erythroid cells **(**Fig. 4B**).** The fibroblast and macrophage cell populations dominated the cells in the EAT from the O-Ex treatment. On the other hand, the B cell, adipocyte, erythroid cell, and mesenchymal cell populations were predominantly comprised of cells from the N-Sed treatment. Most of the T cell cluster was from the N-Ex treatment **(**Fig. 4B**).**

Genes involved in cell adhesion, migration processes, and fatty acid metabolism were modulated in both non-immune and immune cells from N-Sed and O-Sed treatments. All cells except for fibroblasts and macrophages in N-Sed EAT had upregulated expression of fibronectin 1 (*FN1*), which is involved in cell adhesion and migration processes (Dataset S5, Supplemental Fig. 5). On the other hand, fibroblasts in N-Sed EAT showed the highest levels of *ME1*, IQ motif containing GTPase activating protein 2 (*IQGAP2)*, and phospholipid scramblase (*PLSCR)* which are involved in fatty and lipid movement and metabolism. Macrophages in N-Sed EAT had a unique gene expression profile with increased gene expression of only FAT atypical cadherin (*FAT3)* and glutamate ionotropic receptor kainate type 4 (*GRIK4*). O-Sed EAT had highly variable results for DGE among different cell types (Dataset S5, Supplemental Fig. 5). Non-immune cells from O-Sed EAT had upregulated expression of leucine-rich repeat-containing protein (*LRRC43)*, while B cells from O-Sed EAT had upregulated expression of *IQGAP2, CSF1R*, limbic system associated membrane protein (*LSAMP*), and *STAB1* (Dataset S5, Supplemental Fig. 5). T cells had upregulation of 3-oxoacid coA-transferase 1 (*OXCT1*), *TLN2*, pyruvate dehydrogenase kinase 4 (*PDK4*), sorbin and SH3 domain containing 1 (*SORBS1*), growth hormone receptor (*GHR*), diacylglycerol O-acyltransferase 2 (*DGAT2*), phosphodiesterase 3B (*PDE3B*), and *ELOLV6* (Dataset S5, Supplemental Fig. 5). Pathway enrichment analysis showed focal adhesion and AGE-RAGE signaling pathway in diabetic complications were significantly affected in non-immune cells and regulation of actin cytoskeleton and PPAR signaling pathway were involved (Dataset S6).

Genes involved in fatty acid metabolism (*ELOLV6, DGAT2*, lipoprotein lipase (*LPL*), fatty acid binding protein (*AFABP*)) and angiogenesis (matrix gla protein (*MGP*), *GHR*, *CSF1*, microtubule actin crosslinking factor 1 (*MCAF1*)) were upregulated in non-immune cells in O-Ex EAT (Dataset S7, Supplemental Fig. 6). Mesenchymal cells in O-Ex EAT had the most robust upregulation of these genes. Endothelial cells in N-Ex EAT had many upregulated genes such as phospholipase C episilon 1 (*PLCE1*), semaphorin 3 C (*SEMA3C*), basonuclin zinc finger protein 2 (*BNC2*), *PLD1*, cyclin dependent kinase 14 (*CDK14*), glycoprotein M6A (*GPM6A*) and cell adhesion associated, oncogene regulated (*CDON*). The percentage of non-immune cells in EAT expressing genes related to metabolism (i.e. *ELOVL6 and ACACA*) was higher in O-Ex compared to N-Ex pigs (Dataset S7, Supplemental Fig. 6). PPAR signaling pathway and AMPK signaling pathway were significantly affected in non-immune cells (Dataset S8). In N-Ex EAT, macrophages had upregulated *CDON*, EBF transcription factor 1 *(EBF1)*, DLC1 Rho GTPase activating protein *(DLC1)*. In contrast, T and B cells in O-Ex EAT had upregulation of genes similar to non-immune cells in O-Ex EAT (Dataset S7, Supplemental Fig. 6). In the case of immune cells, biosynthesis of unsaturated fatty acids and AMPK signaling pathway were involved (Dataset S8).

Genes involved in fatty acid biosynthesis (*ELOLV6*) and metabolism (*ACACA, ME1*) were highly upregulated in non-immune cells in EAT from O-Ex while an indicator of cytokinesis 9 (*DOCK9*) was highly expressed in O-Sed (Dataset S9, Supplemental Fig. 6). T cells had increased expression of *GHR* in EAT from O-Sed (Supplemental Fig. 5). By contrast, B cells showed higher expression of ACACA and ELOVL6 in EAT from the O-Ex group (Dataset S9, Supplemental Fig. 6). Pathway analysis showed AMPK signaling pathway and PPAR signaling pathway are significantly affected in both B and T cells (Dataset S10).

### Aerobic exercise does not reverse alterations in gene expression in EAT endothelial cells due to coronary artery occlusion and minimally impacts EAT mesenchymal cell gene expression

Sub-clustering of endothelial cells resolved by the four treatment groups resulted in five clusters with separation of the N-Ex group from the rest of the treatment groups (Fig. 4C; Supplemental Fig. 7). Furthermore, the N-Ex group had upregulated gene expression of latent transforming growth factor beta binding protein 2 (*LTBP2*), *PKHD1 Like 1 (PKHD1L1), FERM Domain (FRMD), CDON*, and *GPM6A*, which are all related to cell adhesion and structure (Dataset S11). Upon analysis of the mesenchymal cell cluster based on differential expression of marker genes, four different cell types were identified: endocardial, epicardial fat cells, erythroblasts, and stromal cells (Fig. 4D). Of these identified cell types, most mesenchymal cells were epicardial fat cells. When the subclustered mesenchymal cells were analyzed by treatment group, the only cells associated with N-Ex were epicardial fat cells and erythroblasts (Fig. 4E). The stromal cell subcluster was almost completely comprised of cells from N-Sed. Most endocardial cells were attributed to the O-Sed group. Surprisingly, O-Ex contributed very little to the mesenchymal cell population (Fig. 4E).

## Discussion

This study examined the cellular composition and gene expression of EAT in female pigs in response to aerobic exercise and experimental ischemic heart disease. We applied both bulk RNA-seq and snRNA-seq to gain novel insights into the diversity of cell types in EAT and their responses to exercise and coronary artery occlusion. Bulk RNA-seq revealed that aerobic exercise upregulated pathways related to cell movement and proliferation, particularly in immune cells, myocytes, and fibroblasts. These findings do not completely correspond with those in rodents and humans, which have found that exercise decreases inflammation [31], particularly due to macrophages [32], and increases angiogenesis [33, 34] in subcutaneous (sc) white adipose tissue (WAT). However, most of the studies on the impact of exercise on scWAT have been done in obese subjects and many rodent studies only included males. Interestingly, exercise inhibited pathways related to estrogen signaling and PPARs. This data makes sense as activation of PPAR$\gamma$ stimulates adipogenesis [35] and exercise results in loss of adipose tissue mass [36]. Yet, estrogens are anti-lipogenic [37] and our findings indicate that exercise down-regulates the effects of estrogens in EAT. This finding is the opposite of what we would have expected to find. However, the effect of exercise on estrogen signaling is quite robust as the 2nd most significantly affected network affected by exercise included *ESR1* and *FGFR and* estradiol was a predicted upstream negative regulator of EAT in response to exercise. Based on these findings We chose to apply snRNA-seq to gain a nuanced understanding of the impact of both aerobic exercise and coronary artery occlusion on cell types and gene expression in female EAT. It is important to note that it is common for bulk RNA-seq and snRNA-seq results to often not correspond as whole tissue results can be dominated by a single cell type, which overpowers gene expression that may be occurring in other cell types. That being said, we did find in both data sets that exercise modulated immune cell factors and pathways as well as pathways related to cell movement and proliferation.

A sedentary lifestyle caused the accumulation of B cells and mesenchymal cells in EAT. In obesity, B cells accumulate in WAT and interact with T cells to produce proinflammatory cytokines [38]. Moreover, in scWAT of obese patients, macrophage populations shift from anti-inflammatory M2 cells to pro-inflammatory M1 cells [39]. Surprisingly, short term aerobic exercise decreases M2 macrophage levels in scWAT and visceral (v) WAT of obese male mice [40]. In contrast to what is found in WAT of obese male mice in response to acute exercise, macrophage and T cell numbers were greater in Ex-EAT from female pigs. These results suggest that aerobic exercise impacts inflammation in EAT from females quite differently from scWAT or perhaps highlights the differences between WAT in mice and pigs.

Interestingly, aerobic exercise caused both up-and down-regulation of genes related to lipid metabolism and the inflammatory response, particularly with respect to the interaction of T cells and macrophages in EAT. As aerobic exercise upregulated expression of genes involved in fatty acid synthesis in B cells, but not in T cells or in macrophages, B cells may play a larger role in immunometabolism in EAT than these other two immune cells. This finding is in direct contrast to scWAT where macrophages are one of the main immune cell populations in control of immunometabolism [41]. Mesenchymal stem cells from both humans and mice stimulate regulatory B cell functions via cell-to-cell contact, soluble factors, and extracellular vesicles which lead to anti-inflammatory reactions [42]. In obesity, adipose-derived mesenchymal cells exhibit pro-inflammatory properties, attract inflammatory immune cells, and create an inflammatory microenvironment which causes mesenchymal cell dysfunction [43]. Additionally, B cells accumulate in obese WAT and interact with T cells to produce proinflammatory cytokines [38]. An increased cell-to-cell interaction in the IGF1 pathway between mesenchymal cells, B cells, and T cells in Sed-EAT indicates that a sedentary lifestyle is proinflammatory. In Sed-EAT, T cells interact with mesenchymal cells in the PECAM1 and NCAM pathways which indicates T cells are critical to cell movement and adhesion in Sed-EAT. These findings suggest that adaptive immune cells may serve as a dominant communication “hub” in Sed-EAT and that immune cell function in EAT is quite different from that in other WAT depots.

Coronary artery occlusion interacted with exercise status in EAT to modulate cell number and gene expression. Surprisingly, coronary artery occlusion increased fibrosis and macrophage infiltration in Ex-EAT as opposed to Sed-EAT. Chronic aerobic exercise in males decreases infiltration of M1 macrophages and favors recruitment of M2 macrophages in scWAT [44]. It is possible that coronary artery occlusion counteracts the beneficial effects of chronic exercise on WAT inflammation. It is also possible that the effects of chronic exercise on EAT are different from the effects on other WAT depots. With respect to occlusion status and exercise, N-Ex EAT had large numbers of T cells and endothelial cells. It is not surprising that exercise stimulated increased numbers of endothelial cells in N-Ex EAT, because exercise induces WAT angiogenesis [45]. Our study is the first report of the effects of chronic exercise training on T cell numbers in WAT from any depot. Isolation and immunotyping of T cells from EAT would be the next step to determine the mechanistic implications of this finding. Contrary to what was expected, both N-Sed and O-Sed EAT had upregulation of genes related to cell adhesion and migration. These findings suggest that coronary artery occlusion has minimal effect on these processes in Sed-EAT. However, coronary artery occlusion does result in upregulation of genes related to fatty acid metabolism in T cells from Sed-EAT, which again suggests a critical role for T cells in this EAT.

*ELOVL6*, which is involved in the elongation of long-chain fatty acids [46], was upregulated across all non-immune cell types except mesenchymal cells in Ex-EAT. Interestingly, *ELOVL6* was upregulated in these same cell types as well as B and T cells in O-Ex EAT. *ACACA* and *ACLY*, which are involved in fatty acid synthesis, also were upregulated across all non-immune cell types and B cells in Ex-EAT. However, occlusion status did not affect the expression of these two genes. These findings suggest that exercise may increase the production of fatty acids in most cell types in female EAT. Increased expression of these genes also occurs in scWAT from lean and Roux-en-Y gastric bypass (RYGB) obese patients, which indicates a preference for lipogenesis as opposed to lipolysis in these states [47]. Other studies have found exercise decreases lipogenesis in scWAT of obese female rodents [48] and increases lipolysis in the scWAT of obese and lean men [49]. Increased expression of *FKBP5*, which is a co-chaperone that modulates glucocorticoid action [50], in all immune cell classes in Sed-EAT indicates that the immune system is highly controlled by cortisol in sedentary states. In animal studies, deletion or inhibition of *FKBP5* causes decreased WAT mass and protection against diet-induced weight gain, insulin resistance, and hepatic steatosis [51, 52]. In humans, *FKBP5* expression in abdominal scWAT correlates positively with markers of insulin resistance and type 2 diabetes [53]. Therefore, glucocorticoids seem to have similar effects in all WAT depots, including EAT.

With respect to the interaction of occlusion and exercise, in non-immune cells, especially mesenchymal cells, from O-Ex EAT genes associated with fatty acid metabolism and angiogenesis were upregulated. In contrast, in N-Ex EAT endothelial cells had upregulation of genes involved in cell adhesion and movement. One of the most striking findings in this study was the large number of upregulated genes in T cells in N-EAT, implicating T cells as important to the normal function of EAT in females. There was a five-fold decrease in the number of upregulated genes in T cells in EAT upon coronary artery occlusion. These findings suggest that T cells may be critical to the normal function of EAT in females. Another remarkable finding was the separation of endothelial cells in N-Ex EAT from endothelial cells in EAT from all other treatments. Given that the gene signature of endothelial cells in O-Ex EAT is similar to the gene signatures of O-Sed and N-Sed EAT suggests that coronary artery occlusion blocks the potential beneficial effects of aerobic exercise on endothelial cells in EAT. Endothelial cells are essential for vascular function, the delivery of nutrients and oxygen, and removal of wastes from EAT [54]. Therefore, this finding coupled with the fact that O-Ex EAT has more fibrosis and macrophage infiltration indicates that coronary artery occlusion in females is extremely detrimental to the health of the surrounding EAT. Moreover, chronic aerobic exercise is unable to counteract these deleterious effects. Sub-clustering of mesenchymal cells found that most of the cells in this population were from Sed-EAT. In high fat diet fed mice, mesenchymal stem cells can differentiate into adipocytes but exercise reverses this effect [55]. Our findings with respect to mesenchymal cells correspond with study and support the ability of exercise to limit differentiation of the EAT mesenchymal cell pool into epicardial fat cells.

## Conclusions

In conclusion, this study highlights that chronic aerobic exercise increases interaction between immune, mesenchymal, and endothelial cells in EAT from female pigs. Furthermore, genes related to cell adhesion and metabolism in these cell types and adipocytes and smooth muscle cells are upregulated in response to exercise. T cells appear to be critical to the normal function of female EAT, whereas B cells are the immune cell class in female EAT most sensitive to aerobic exercise. Surprisingly, chronic aerobic exercise is minimally effective at reversing alterations in gene expression in endothelial and mesenchymal cells in EAT surrounding occluded coronary arteries in female pigs. These findings lay the foundation for future work focused on the mechanistic impact of exercise on various cell types in EAT from women.

### Electronic supplementary material

Below is the link to the electronic supplementary material.

**Supplementary Material 1: Supplemental Figure S1:** Violin plots showing post-exercise upregulation of gene expression associated with IGF signaling, NCAM signaling and PECAM1 signaling pathways grouped by cell type across each treatment

**Supplementary Material 2: Supplemental Figure S2:** Total number of upregulated and downregulated genes between (A) exercised and sedentary groups and (B) occluded and non-occluded coronary arteries in epicardial adipose tissue. The intensity of the red color corresponds to the number of DEGs, with red being the largest number of DEGs (1500) and light pink to white being the smallest number of DGE (0). The length of the grey bar next to each cell type corresponds to the total number of upregulated or downregulated DEGs by cell type. The largest numbers of genes were altered in T cells and endothelial cells. P < 0.05 and Average Log Fold Change > 1

**Supplementary Material 3: Supplemental Figure S3:** Expression of markers for cell types from epicardial adipose tissue from female pigs. Yellow is upregulated and magenta is downregulated expression level of the top 42 genes expressed in all cell types. As this study was conducted in porcine epicardial adipose tissue, many of the genes have not been identified by homologous name and function to human genes and, as such, are shown by their Ensembl Stable Id

**Supplementary Material 4: Supplemental Figure S4:** Dot plots of differential gene expression within each cell type in epicardial adipose tissue from exercised and sedentary female pigs. Pink represents upregulated and gray represents downregulated transcript expression level. The dot size represents the percentage of cells expressing the gene in each cell type in exercise-trained and sedentary groups. Ex: exercise-trained; Sed: sedentary

**Supplementary Material 5: Supplemental Figure S5:** Dot plots of the differential gene expression within each cell type in epicardial adipose tissue surrounding occluded or non-occluded coronary arteries from sedentary female pigs. Pink represents upregulated and gray represents downregulated transcript expression level. The size of the dot represents the percentage of cells expressing the gene in each cell type in sedentary non-occluded and occluded groups. N-Sed: non-occluded sedentary. O-Sed: occluded sedentary

**Supplementary Material 6: Supplemental Figure S6:** Dot plots of the differential gene expression within each cell type in epicardial adipose tissue surrounding occluded or non-occluded coronary arteries from exercise-trained female pigs. Pink represents upregulated and gray represents downregulated transcript expression level. The size of the dot represents the percentage of cells expressing the gene in each cell type in exercised non-occluded and occluded groups. N-Ex: non-occluded exercise-trained. O-Ex: occluded exercise-trained

**Supplementary Material 7: Supplemental Figure S7:** Expression of top transcript markers specifically for endothelial cells in different subclusters found in single nucleus sequencing of epicardial adipose tissue from exercise-trained and sedentary female pigs. Yellow is upregulated and magenta is downregulated expression level of the top 25 genes expressed in all cell types. As this study was conducted in porcine epicardial adipose tissue, many of the genes have not been identified by homologous name and function to human genes and, as such, are shown by their Ensembl Stable Id

**Supplementary Material 8:** Supplementary Table 1

**Supplementary Material 9:** Supplementary Table 2

**Supplementary Material 10:** Supplementary Table 3

**Supplementary Material 11:** Supplementary Table 4

**Supplementary Material 12:** Dataset S1

**Supplementary Material 13:** Dataset S2

**Supplementary Material 14:** Dataset S3

**Supplementary Material 15:** Dataset S4

**Supplementary Material 16:** Dataset S5

**Supplementary Material 17:** Dataset S6

**Supplementary Material 18:** Dataset S7

**Supplementary Material 19:** Dataset S8

**Supplementary Material 20:** Dataset S9

**Supplementary Material 21:** Dataset S10

**Supplementary Material 22:** Dataset S11

**Supplementary Material 23:** Supplemental Methods

## Data Availability

Bulk RNA-seq and snRNA-seq data are deposited into the BioSample database under accession number GSE246709. Supplementary Data for the present findings are available within Supporting Information files. Scripts for data processing and downstream analyses are available through GitHub at https://github.com/Xenon8778/Pig_EAT_scRNAseq.

## Abbreviations

ACACA: acetyl-coA carboxylase alpha
ACAT1: acetyl-coA acetyl transferase 1
ACOX1: acyl-coA oxidase 1
ACYL: acyl-coA
ADGRD1: adhesion G protein-coupled receptor D1
ADGRL4: adhesion G protein-coupled receptor
ADIPOR2: adiponectin receptor 2
AFABP: fatty acid binding protein
APBB1IP: ameloid beta precursor protein binding family B member 1 interacting protein
APOA1: apolipoprotein A1
APOE: apolipoprotein E
AR: androgen receptor
BANK1: B cell scaffold protein with ankyrin repeats 1
BLNK: B cell linker
BNC2: basonuclin zinc finger protein 2
CAD: coronary artery disease
CCN4: cellular communication network factor 4
CD93: CD93 molecule
CD136: CD136 molecule
CDK14: cyclin dependent kinase 14
CDON: cell adhesion associated oncogene regulated
CKAP2: cytoskeleton associated protein 2
CREB: cAMP response element binding protein
CSF1R: colony stimulating factor 1 receptor
CSRP1: cysteine and glycine rich protein 1
CYYR1: cysteine and tyrosine rich 1
DGAT2: diacylglycerol O-acyltransferase 2
DGE: differential gene expression
DLC1: DLC1 Rho GTPase activating protein
DOCK9: dedicator of cytokinesis 9
EAT: epicardial adipose tissue
EBF1: EBF transcription factor 1
ECT2L: epithelial cell transforming 2 like
ELOLV6: ELOLV fatty acid elongase 6
ESR1: estrogen receptor alpha
ESRRG: estrogen related receptor gamma
Ex: exercised
FAK: focal adhesion kinase
FAT3: FAT atypical cadherin
FHIP1A: FHF complex subunit HOOK interacting protein 1 A
FIS1: mitochondrial fission 1
FKBP5: FKBP prolyl isomerase 5
FLT1: fms related receptor tyrosine kinase
FN1: fibronectin 1
FRMD: FERM domain
GHR: growth hormone receptor
GLUL: glutamate-ammonia ligase
GPCR: G-protein coupled receptor
GPIHBP1: glycosylphosphatidylinositol anchored high density lipoprotein binding protein 1
GPM6A: glycoprotein M6A
GRIK4: glutamate ionotropic receptor kainite type 4
GSN: gelsolin
HADH: hydroxyacyl-coA dehydrogenase
HBEGF: heparin binding EGF-like growth factor
IL-13: interleukin 13
IL-4: interleukin 4
IGF: insulin-like growth factor
IGFBP2: insulin-like growth factor binding protein 2
IKZF3: IKAROS family zinc finger 3
IQGAP2: IQ motif containing GTPase activating protein 2
KRT7: keratin 7
KRT18: keratin 18
LDB2: LIM binding domain 2
LPL: lipoprotein lipase
LRRC43: leucine-rich repeat-containing protein
LSAMP: limbic system associated membrane protein
LTBP2: latent transforming growth factor beta binding protein 2
N: non-occluded
NCAM: neural cell adhesion molecule
NIH: National Institutes of Health
NNAT: neuronatin
MCAF1: microtubule actin crosslinking factor 1
MGP: matrix gla protein
MMRN1: multimerin 1
MUSK: muscle associated receptor tyrosine kinase
O: occluded
OXCT1: 3-oxoacid coA-transferase 1
PARD6G: par-6 family cell polarity regulator gamma
PDE3B: phosphodiesterase 3B
PDK4: pyruvate dehydrogenase kinase 4
PDXC1: plexin domain containing 1
PECAM1: platelet endothelial cell adhesion molecule 1
PFKFB3: 6-phosphofructo-2-kinase/fructose-2,6-biphophatase 3
PIM2: pim-2 proto-oncogene
PKHD1L1: PKHD1 like 1
PLCE1: phospholipase C episilon 1
PLD1: phospholipase D1
PLS1: plastin 1
PLSCR: phospholipid scramblase
POSTN: periostin
PTPRB: protein tyrosine phosphatase receptor type B
PTPRC: protein tyrosine phosphatase receptor type C
PROX1: prospero homeobox 1
RAMP2: receptor activity modifying protein 2
REEP1: receptor accessory protein 1
RGS5: regulator of G protein signaling
RNAseq: bulk RNA sequencing
SCARA5: scavenger receptor class A member 5
SCD: stearoyl-CoA desaturase
Sed: sedentary
SEMA3C: semaphorin 3 C
SEMA6A: semaphorin 6 A
SERPINE 1: serpine family E number 1
SKAP1: Src kinase associated phosphoprotein 1
SLC5A6: solute carrier family 5 member 6
snRNA-seq: single-nuclei RNA sequencing
SORBS1: sorbin and SH3 domain containing 1
SPON1: spondin 1
STAB1: stabilin
SYK: spleen associated tyrosine kinase
SYNPO2: synaptopodin 2
TLN2: talin 2
TPT1: tumor protein translastionally-controlled 1
VAMP3: vesicle associated membrane protein 3
VAV3: vav guanine nucleotide exchange factor 3
WAT: white adipose tissue
ZBTB7C: zinc finger binding domain containing 7 C
