# Supplementary figures and images for: Single-nucleus transcriptomics of epicardial adipose tissue from female pigs reveals effects of exercise training on resident innate and adaptive immune cells
Source: Cell Commun Signal. 2024 Apr 26;22:243. doi: 10.1186/s12964-024-01587-w (PMC11046969; doi:10.1186/s12964-024-01587-w)

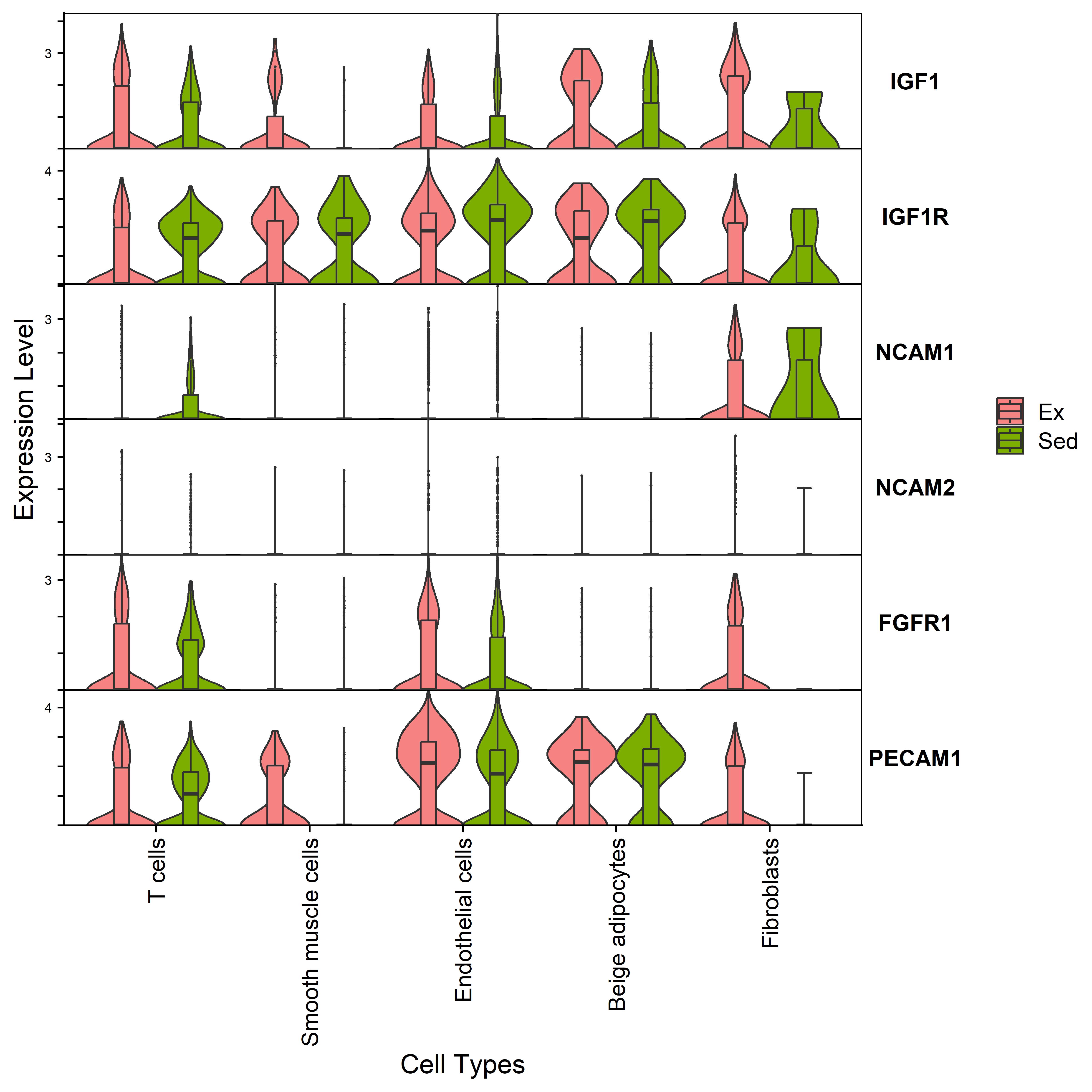

Supplement: Supplementary file 1 — Supplementary Material 1: Supplemental Figure S1: Violin plots showing post-exercise upregulation of gene expression associated with IGF signaling, NCAM signaling and PECAM1 signaling pathways grouped by cell type across each treatment [file 12964_2024_1587_MOESM1_ESM.jpeg]

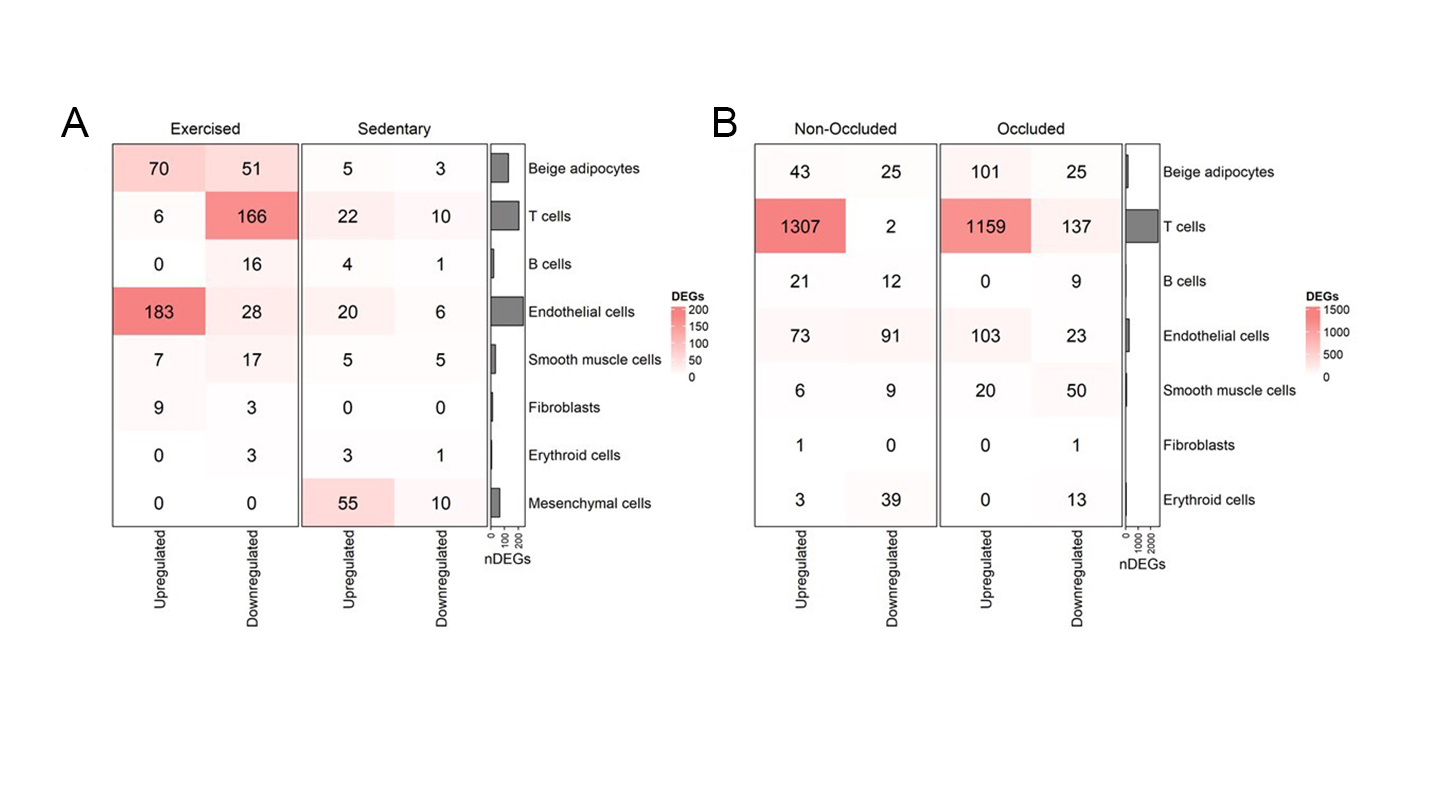

Supplement: Supplementary file 2 — Supplementary Material 2: Supplemental Figure S2: Total number of upregulated and downregulated genes between (A) exercised and sedentary groups and (B) occluded and non-occluded coronary arteries in epicardial adipose tissue. The intensity of the red color corresponds to the number of DEGs, with red being the largest number of DEGs (1500) and light pink to white being the smallest number of DGE (0). The length of the grey bar next to each cell type corresponds to the total number of upregulated or downregulated DEGs by cell type. The largest numbers of genes were altered in T cells and endothelial cells. P < 0.05 and Average Log Fold Change > 1 [file 12964_2024_1587_MOESM2_ESM.jpg]

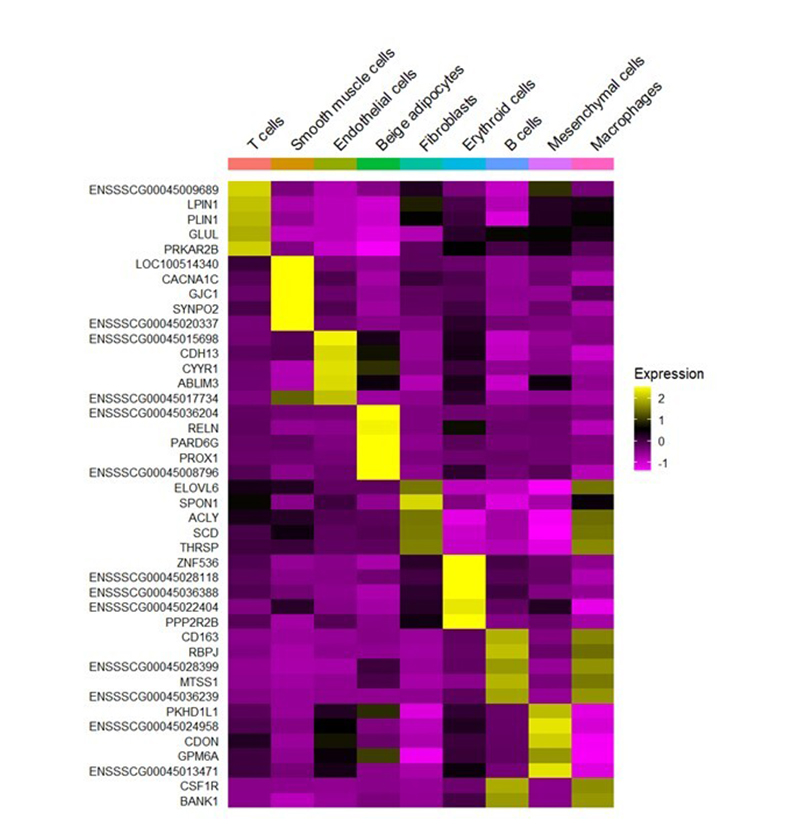

Supplement: Supplementary file 3 — Supplementary Material 3: Supplemental Figure S3: Expression of markers for cell types from epicardial adipose tissue from female pigs. Yellow is upregulated and magenta is downregulated expression level of the top 42 genes expressed in all cell types. As this study was conducted in porcine epicardial adipose tissue, many of the genes have not been identified by homologous name and function to human genes and, as such, are shown by their Ensembl Stable Id [file 12964_2024_1587_MOESM3_ESM.jpg]

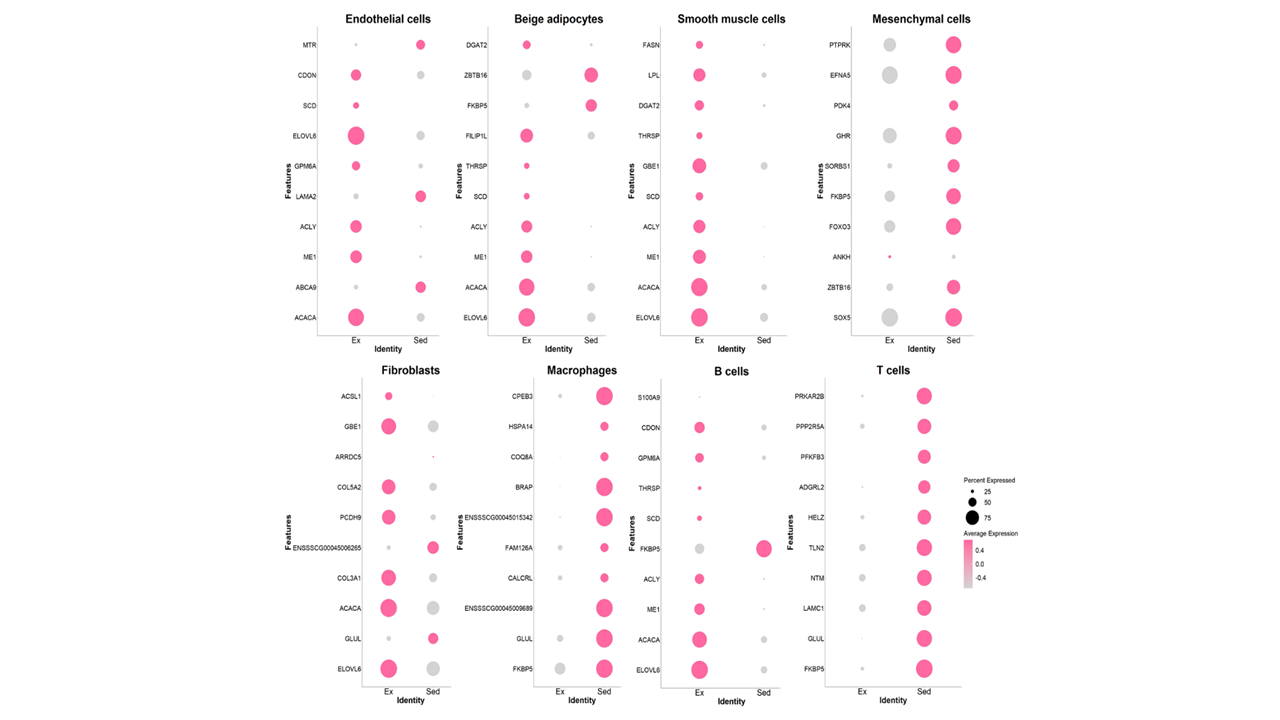

Supplement: Supplementary file 4 — Supplementary Material 4: Supplemental Figure S4: Dot plots of differential gene expression within each cell type in epicardial adipose tissue from exercised and sedentary female pigs. Pink represents upregulated and gray represents downregulated transcript expression level. The dot size represents the percentage of cells expressing the gene in each cell type in exercise-trained and sedentary groups. Ex: exercise-trained; Sed: sedentary [file 12964_2024_1587_MOESM4_ESM.tiff]

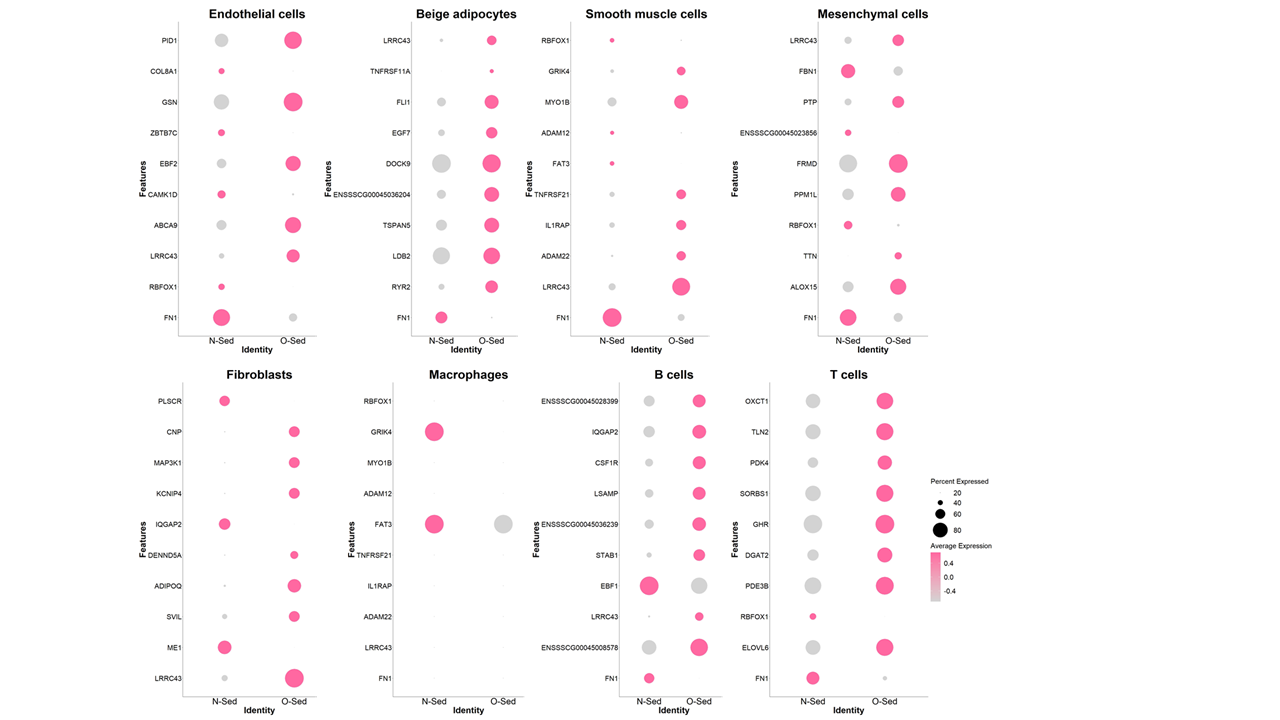

Supplement: Supplementary file 5 — Supplementary Material 5: Supplemental Figure S5: Dot plots of the differential gene expression within each cell type in epicardial adipose tissue surrounding occluded or non-occluded coronary arteries from sedentary female pigs. Pink represents upregulated and gray represents downregulated transcript expression level. The size of the dot represents the percentage of cells expressing the gene in each cell type in sedentary non-occluded and occluded groups. N-Sed: non-occluded sedentary. O-Sed: occluded sedentary [file 12964_2024_1587_MOESM5_ESM.tiff]

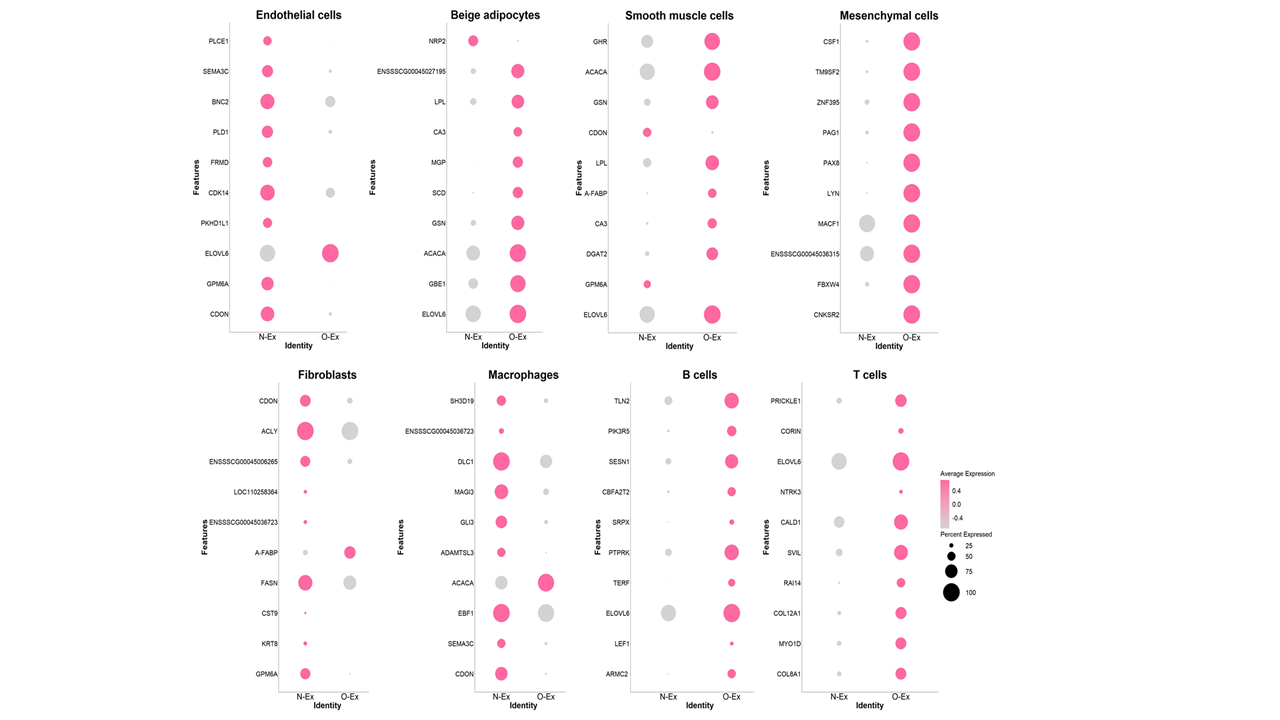

Supplement: Supplementary file 6 — Supplementary Material 6: Supplemental Figure S6: Dot plots of the differential gene expression within each cell type in epicardial adipose tissue surrounding occluded or non-occluded coronary arteries from exercise-trained female pigs. Pink represents upregulated and gray represents downregulated transcript expression level. The size of the dot represents the percentage of cells expressing the gene in each cell type in exercised non-occluded and occluded groups. N-Ex: non-occluded exercise-trained. O-Ex: occluded exercise-trained [file 12964_2024_1587_MOESM6_ESM.tiff]

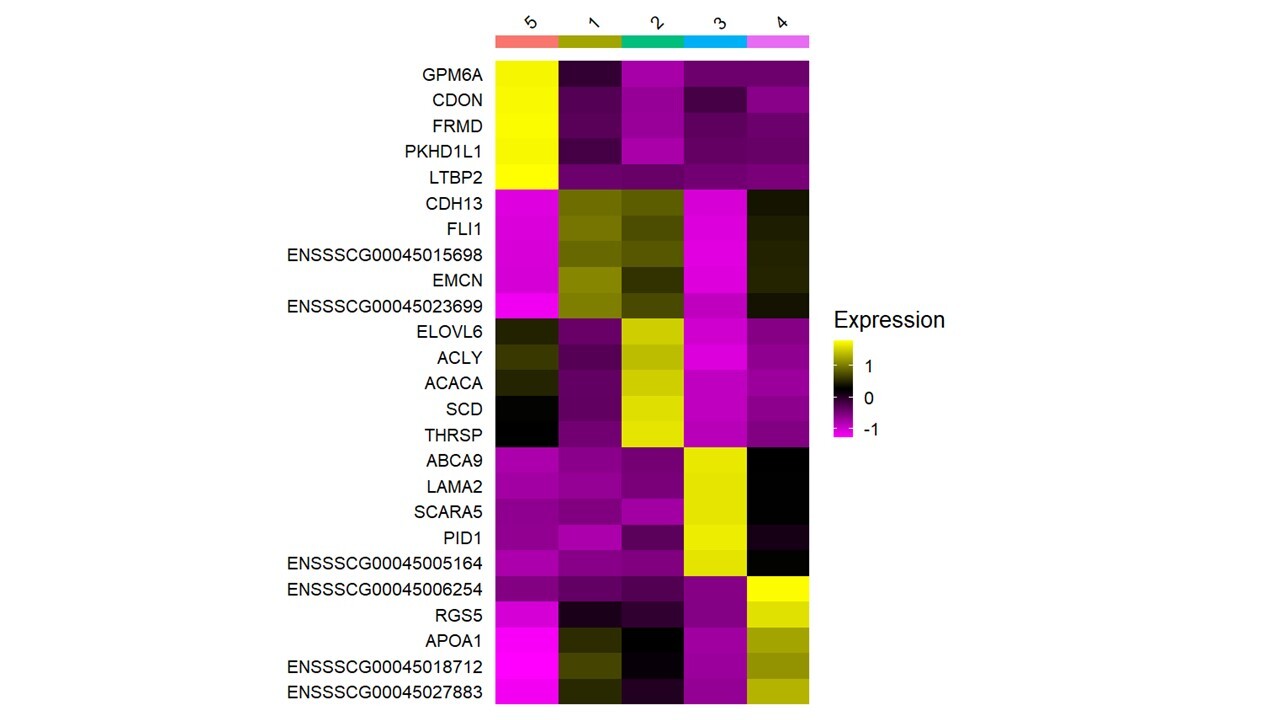

Supplement: Supplementary file 7 — Supplementary Material 7: Supplemental Figure S7: Expression of top transcript markers specifically for endothelial cells in different subclusters found in single nucleus sequencing of epicardial adipose tissue from exercise-trained and sedentary female pigs. Yellow is upregulated and magenta is downregulated expression level of the top 25 genes expressed in all cell types. As this study was conducted in porcine epicardial adipose tissue, many of the genes have not been identified by homologous name and function to human genes and, as such, are shown by their Ensembl Stable Id [file 12964_2024_1587_MOESM7_ESM.jpg]
